# Parent-of-origin effects of phosphatidyl inositol-bisphosphate hydrolysis pathway genes on type 2 diabetes and the modification effect by obesity

**DOI:** 10.3389/fendo.2025.1705584

**Published:** 2025-11-27

**Authors:** Yinxi Tan, Hexiang Peng, Siyue Wang, Poning Hu, Yi Zheng, Haodong Zhang, Huangda Guo, Yixin Li, Hanyu Zhang, Yiqun Wu, Xueying Qin, Jing Li, Tao Wu, Dafang Chen, Yonghua Hu, Mengying Wang

**Affiliations:** 1 Department of Nutrition and Food Hygiene, School of Public Health, Peking University, Beijing, China; 2 Department of Epidemiology and Health Statistics, Xiangya School of Public Health, Central South University, Changsha, China; 3 Department of Epidemiology and Biostatistics, School of Public Health, Peking University, Beijing, China; 4 Key Laboratory of Epidemiology of Major Diseases (Peking University), Ministry of Education, Beijing, China

**Keywords:** parent-of-origin effect, case-parents triad, type 2 diabetes, interaction analysis, cohort study

## Abstract

**Objective:**

To investigate the parent-of-origin effects (POEs) of genes in the phosphatidyl inositol-bisphosphate (PIP2) hydrolysis pathway on type 2 diabetes (T2D) and preliminarily assess whether environmental factors may modify these effects.

**Methods:**

Based on data from an ongoing family-based cohort in Beijing, genetic information of 162 individuals from 53 case-parent triads was used to examine the POE of single nucleotide polymorphisms (SNPs) in the PIP2 pathway on T2D using maximum likelihood estimation based on a log-linear model. Stratified analyses were performed to assess the potential modification of POE by environmental factors, including smoking, drinking, and body mass index (BMI). Further enrichment analysis was conducted based on the POE results.

**Results:**

A total of 214 SNPs from the PIP2 hydrolysis pathway had nominally significant (*P* < 0.05) POE on T2D, among which rs199684931 (RR_m_/RR_p_ = 0.28, *P*_interaction_ = 0.03), rs4750491 (RR_m_/RR_p_ = 4.67, *P*_interaction_ = 0.04), rs1090705 (RR_m_/RR_p_ = 0.21, *P*_interaction_ = 0.04), rs9663645 (RR_m_/RR_p_ = 4.75, *P*_interaction_ = 0.04), and rs200488869 (RR_m_/RR_p_ = 4.75, *P*_interaction_ = 0.04) from *PRKCQ* exhibited POE–BMI interactions. Specifically, maternal POE was reduced for rs199684931 and rs1090705 in individuals with higher BMI levels, while it increased for rs4750491, rs9663645, and rs200488869 in higher BMI groups. Additionally, 72 of the 214 significant POE SNPs were recognized as methylation quantitative trait loci, hinting at a possible role in regulation. The enrichment analysis validated these findings and the role of the genes in lipid metabolism.

**Conclusion:**

The current study provides a preliminary hint that SNPs in the PIP2 pathway genes may exhibit POE on T2D, contributing to its heritability. Notably, five SNPs in the *PRKCQ* gene demonstrated a potential interaction between POE and BMI on T2D. Further research is necessary to explore the underlying molecular mechanisms and to validate these findings in larger and independent populations.

## Introduction

Type 2 diabetes (T2D) is a leading cause of global death and disability, the prevalence of which has risen dramatically, with projections estimating that more than 1.31 billion people will have it by 2050 (1, 2). Both genetic and environmental risk factors contribute to the etiology of T2D, and genome-wide association studies (GWASs) (3, 4) have identified numerous genetic loci associated with T2D. However, these GWASs still lack detailed explanations regarding the heritability of T2D (5). Notably, specific loci showing parent-of-origin effects (POEs) on T2D have been identified, suggesting that the genetic risk may depend on whether the allele is inherited from the maternal or paternal side (6). POE might be attributed to genomic imprinting (7) and provide a new vision on the genetic etiology of T2D (5). Previously, POE on T2D has been observed in several genes including *KLF14*, *MOB2*, and *KCNQ1* (6, 8). For instance, intronic variants of *KCNQ1* affect the susceptibility of T2D when inherited from the mother (9). Moreover, DNA methylation may be one of the important forms of epigenetic modification involved in the achievement and maintenance of POE in T2D (10). Despite these findings, the investigation of POE in T2D remains underexplored, shedding light on a potential gap in understanding the genetic mechanisms underlying this disease. Studying POE and its interaction with environmental factors could better identify parent-specific epigenetic risk intervention targets, providing precise prevention methods in public health practice (11–13).

Hydrolysis of phosphatidyl inositol-bisphosphate (PIP2) by phospholipase C (PLC) produces diacylglycerol (DAG) and inositol triphosphate (IP3) (14), with the effects on signaling cascades, molecular interactions, and altered cellular functions. PIP2 is an important lipid molecule in the insulin signaling pathway (15) and is involved in KCNQ1 activation (16). It is reported that PIP2-PLC activity increased in the liver of obese patients with T2D (17). The pathway “effects of PIP2 hydrolysis” (the PIP2 pathway) highlights the downstream biological impacts of PIP2 breakdown, focusing on its products of DAG and IP3. DAG contributes to insulin resistance in T2D by activating PKC (18), which disrupts insulin signaling pathways. Meanwhile, IP3-induced calcium release can trigger β-cell dysfunction and apoptosis, impairing insulin secretion and exacerbating the disease (19). PIP2 serves as the essential substrate for phosphatidylinositol-3 kinase (PI3K) to generate phosphatidyl inositol triphosphate (PIP3) (20), the lipid second messenger that initiates the downstream Akt signaling cascade. As another potential pathway, the elevated expression of a kinase, Src-Homology Inositol Phosphatase-2 (SHIP2) that hydrolyzes PIP3 to PIP2, could reduce insulin signaling and contribute to insulin resistance in T2D (21). Therefore, investigating the PIP2 pathway provides a more integrated and mechanistic perspective for understanding T2D pathogenesis, as it captures the upstream signaling event that concurrently governs multiple downstream pathological processes.

Lipid metabolism has been associated with POE of other specific genes (22, 23). Genes on the PIP2 pathway were reported to be associated with T2D before, including *DAGLB* (24), *DGKB* (25), *DGKI* (26), *ITPR2* (27), etc. However, whether these genes on this pathway could influence T2D risk through POE remains unclear. Hence, to our knowledge, the current study was the first to explore the associations between the POE of T2D and genes on the PIP2 pathway.

The family-based study design has distinct strengths in POE investigations for better control of the influence of population stratification and greatly improves the efficacy of gene research. Under this ideal study design, we conducted a study using T2D families in the Chinese population to explore whether the SNPs in the PIP2 pathway could influence T2D by POE.

## Methods

### Study design and participants

This study was based on an ongoing family-based cohort established in Fangshan District, Beijing, China. Briefly, this cohort focused on the etiology research on chronic diseases in the northern Han Chinese population, with intact genetic information to decipher the genetic predisposition. The master protocol was published elsewhere (28), with a detailed description of the establishment and settings of this cohort.

After excluding participants without intact family information or genetic data, a total of 54 triads with all offspring with a T2D diagnosis were included to explore the POE of SNPs in the PIP2 pathway associated with T2D. Among the 162 participants from 54 triads, 110 were T2D cases and 52 were controls.

### Measurements

All participants in this study were privately interviewed and examined physically, with biospecimens collected by local medical professionals.

### Genetic data

Blood samples were preprocessed and stored at −80 °C at the examination center. Through trained technicians and the Infinium Asian Screening Array-24 v1.0 BeadChip, genetic DNA was extracted by a LabTurbo 496-Standard System (TAIGEN Bioscience Corporation, Taiwan, China), with the DNA purity and concentration measured through ultraviolet spectrophotometry. The methylation test was conducted based on the Illumina Infinium Methylation EPIC BeadChip (935K). The level of methylation expressed by *β* was calculated as follows:


$$\beta =\;\frac{Max(M,\;0)}{Max\;(M,\;0)+Max(U,\;0)+100}$$

Where *M* represents the signal intensity of fully methylated sites, and *U* represents the signal intensity of unmethylated sites. The *β* value ranges from 0 to 1, indicating the continuous variable of methylation level.

All 27 genes included in the PIP2 pathway were recorded from the PathCards database (https://pathcards.genecards.org/, accessed on 23 October 2025), with the gene position data downloaded from the NCBI database (https://www.ncbi.nlm.nih.gov, accessed on 23 October 2025). Each sample underwent quality control (QC) to ensure data reliability, including a variant call rate exceeding 95%, consistency between genotyped and reported sex, heterozygosity within 3 standard deviations (SDs), agreement between T2D status and the investigated kinship, less than 5% Mendelian errors, and no significant deviation in principal component analyses of ancestral background. QC was also applied to each call set, requiring a sample call rate above 95%, Hardy–Weinberg equilibrium (*P >*1 × 10^−6^), a minimum allele frequency (MAF) greater than 1%, and fewer than 10% Mendelian errors. These QC procedures were all conducted using PLINK 1.9 software (https://www.cog-genomics.org/plink/). A total of 9,191 SNPs in the 27 genes in the PIP2 pathway were included in the current analysis.

### Outcome definition

In this study, T2D was defined by self-reported records or abnormal glycemic markers, including fasting blood glucose ≥7.0 mmol/L, or HbA_1c_ ≥6.5%, or a history of drug use for diabetes, with clinical physicians confirming the diagnosis. All prevalent cases at baseline and incident cases during the cohort follow-up were included in the analysis.

### Covariates

Demographics, lifestyles, and medical records of all participants were collected by trained investigators at baseline. Anthropometric tests included height (meters) and weight (kilograms), with all plasma samples collected. Body mass index (BMI) is calculated as weight (kg)/height (m)^2^, where obesity is defined as having a BMI ≥28 kg/m^2^.

Synchronized by children in each family, the condition of smoking (yes/no), drinking (yes/no), and obesity (≥28/<28 kg/m^2^) defined by BMI were recognized as environmental factors, all by two categorized classifications in this study. All environmental factor levels of offspring were taken to explore whether the POE would be modified by the offspring’s characteristics.

### Statistical analysis

The POE analysis on the case-parents triads was analyzed by maximum likelihood estimation based on a log-linear model, with markers or haplotype frequencies estimated by “Haplin” package in R (Version 3.4.1) (29, 30). To be specific, a single and multi-allelic locus with corresponding population allele frequencies p_1_, p_2_, p_3_…p_k_ was coded as K alleles A_1_, A_2_, A_3_…A_k_. M, F, and C symbolize the genotypes of mother, father, and child in each triad, so the full triad is denoted as (M, F, C) = (A_i_A_j_, A_k_A_l_, A_j_A_l_). Assuming the second allele from the parents was transmitted to the child for convenience, the full triad could be described by the mating type (M, F) = (A_i_A_j_, A_k_A_l_). As all children in this study are cases and D denotes the child’s case, the model for POE and maternal effects is written as follows:


$$\text{P}(\text{D}|{\text{A}}_{\text{i}}{\text{A}}_{\text{j}},{\text{ A}}_{\text{k}}{\text{A}}_{\text{l}})=\text{B}\cdot {\text{RR}}_{\text{M},\text{j}}{\text{RR}}_{\text{F},\text{j}}{\text{RR}}_{\text{jl}}^{*}\times {\text{RR}}_{\text{i}}^{\left(\text{M}\right)}{\text{RR}}_{\text{j}}^{\left(\text{M}\right)}{\text{RR}}_{\text{ij}}^{(\text{M})*}$$

where RR_M,j_ and RR_F,j_ are the risk increase or decrease associated with the allele A_j_ and A_l_, relative to the baseline risk level B, depending on whether this allele is transmitted from the mother or the father. RR_i_^(M)^ is the relative risk associated with allele A_i_ transmitted from the mother, and so is RR_j_^(M)^. RR_jl_^*^ and RR_ij_^(M)*^ are included to deviate the risk of homozygous individuals from the expectations of the multiplicative model. Meanwhile, an expectation–maximization algorithm was used to estimate the missing markers and haplotype information, to better enhance the estimation effect of POE by haplotypes. POE–environment interaction tests were conducted based on the former POE results, with stratification by each environmental factor level. The characteristics of all SNPs analyzed in this study are provided in **Supplementary Table S1**. False discovery rate (FDR) correction was adapted to both the main analysis and the interaction analysis by the R stats “p.adjust,” based on the calculation algorithm raised by Benjamini and Hochberg, controlling the false discovery rate (31).

Moreover, since the POE of T2D may be driven by DNA methylation (32, 33), we further identified the intersections between the SNPs that exhibited significant results in the POE analysis and the methylation quantitative trait loci (mQTLs) based on the Chinese population (34). Also, analysis of the association between the CpG sites near the top 10 significant SNPs and T2D was conducted in a subset of 99 participants with complete mQTL data.

For SNPs with *P* < 0.05 in the POE analysis, Gene Ontology (GO) enrichment analysis was conducted to further annotate these genes’ functions in the molecular and cellular aspects. The g:GOSt website (https://biit.cs.ut.ee/gprofiler/gost) (35) was applied to identify significantly enriched gene ontology terms among a list of novel SNP annotated genes by the well-proven cumulative hypergeometric test. The *P*-values were calculated based on the cumulative hypergeometric distribution, with multiple testing conducted by a Set Counts and Sizes (g: SCS) method (35).

A sensitivity analysis was performed by classifying offspring sex. A *P*-value < 0.05 was considered statistically significant in the POE and POE–environment interaction analyses.

## Results

### Association study and POE analysis of SNPs in PIP2 pathway genes with T2D

**Figure 1:**
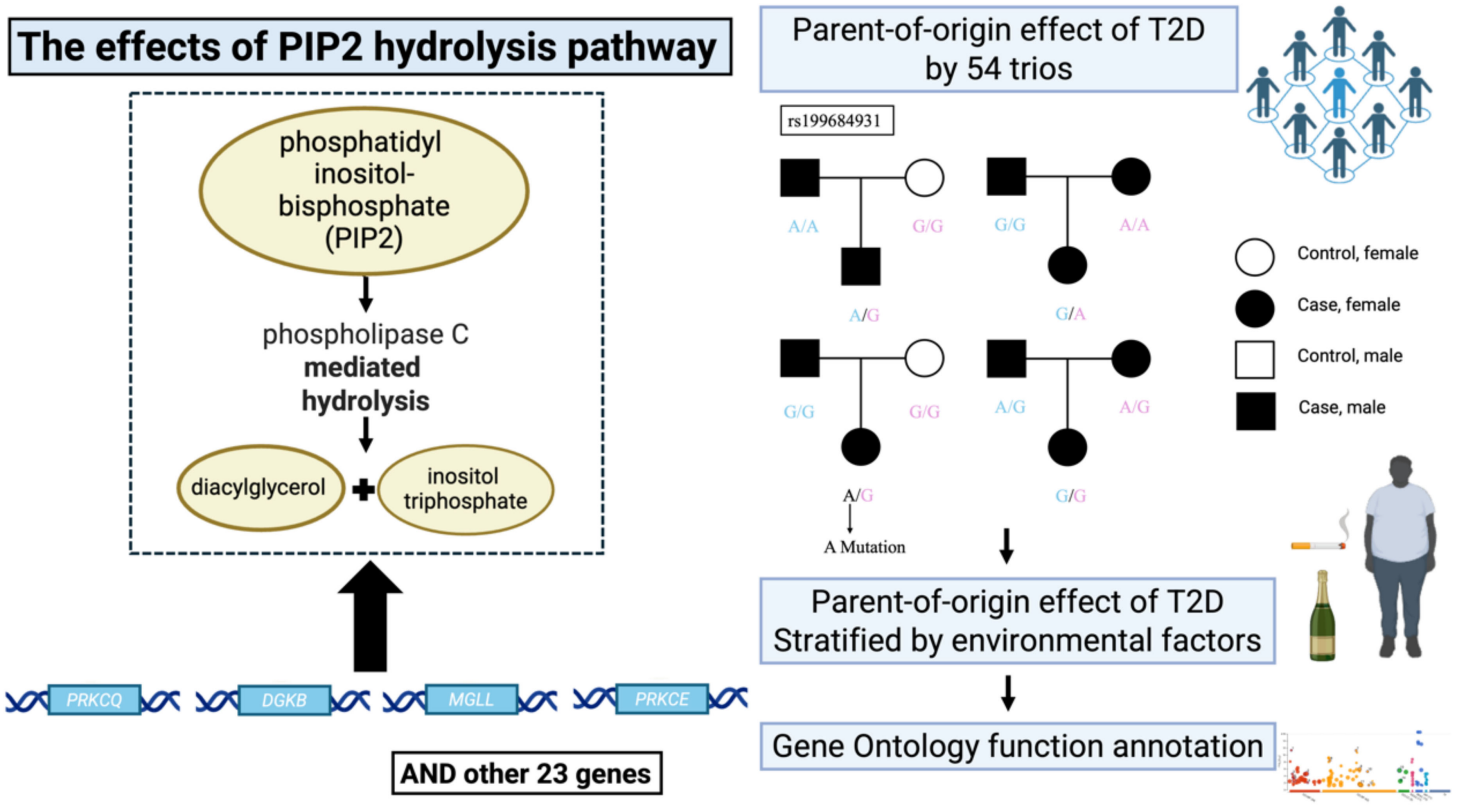
Schematic design of the study. Figure was created using Biorender (https://www.biorender.com).

The POE analysis included 54 triads, with six families including two offspring, each allocated to a different trio for analysis. The schematic composition of the triad is illustrated in **Figure 1**. A total of 9,191 SNPs in this pathway had been examined for POE on T2D, among which 214 showed *P* < 0.05 (the top 10 SNPs are shown in **Table 1**). However, due to the limited sample size, the *P*-values after FDR correction were over 1, none of which passed the FDR correction, presenting them as candidate loci for future investigation. Specifically, 53 significant results in *PRKCQ*, 50 in *DGKB*, 36 in *MGLL*, 34 in *PRKCE* (**Supplementary Figure S1**), and a few significant loci in *DGKH*, *DAGLB*, *ITPR1*, *ITPR2*, *DGKQ*, *PRKCH*, *RASGRP2*, *DGKD*, and *DGKG* (see **Supplementary Table S2**) were observed. The top 10 significant loci came from *ITPR1*, *PRKCE*, and *PRKCQ* genes, as shown in **Table 1**. For example, rs6798160 in the *ITPR2* gene showed the lowest *P*-value in the POE analysis, with an estimated POE by RR_m_/RR_f_ of 0.11 (95% CI: 0.03–0.38), revealing that the SNP had a much lower impact when inherited from maternal (RR_m_) compared to that inherited from the paternal (RR_f_) on T2D.

**Table 1:** The results of the most significant loci of POE on T2D.

Gene	SNP	Estimate of POE, the ratio RR_m_/RR_f_	Lower	Upper	*P*-value of POE
*ITPR1*	rs6798160	0.11	0.03	0.38	**6.34 × 10^−4^**
*PRKCE*	rs7572708	7.42	2.19	25.14	**0.001**
*PRKCQ*	rs12774213	0.18	0.06	0.51	**0.001**
*PRKCE*	rs12466823	7.59	2.15	25.87	**0.002**
*PRKCE*	rs12473396	0.18	0.06	0.54	**0.002**
*PRKCQ*	rs7079678	0.19	0.07	0.55	**0.002**
*PRKCQ*	rs34851049	0.19	0.07	0.55	**0.002**
*PRKCE*	rs62127222	0.19	0.07	0.55	**0.002**
*PRKCQ*	rs7101262	0.19	0.07	0.55	**0.002**
*PRKCQ*	rs4750491	4.67	1.71	12.34	**0.002**

Bold font symbolized results with statistical significance.

The triad graph illustrates the design of the POE analysis: The children inherit each allele from the father (left blue allele) and mother (right pink allele), respectively. The left part shows the 27 genes in the PIP2 pathway, with four displayed genes holding the most significant loci of POE in T2D.

### Characteristics of the genotypes of the significant loci of POE in the 54 triads

Among the 54 case children included in this study, some individuals can determine which parent each risk allele of the heterozygous SNPs originates from, as shown in **Table 2**. It was observed that the parent contributing the risk allele often aligned with the POE. Specifically, 33 case children were analyzed for rs199684931, where the fathers contributed more of the risk allele G. The POE analysis of rs199684931 also indicated a paternal effect.

**Table 2:** Characteristics of the risk allele of POE on T2D among the case children.

SNP (cases)	Risk allele	Allele inherited from the	
		Father	Mother
rs199684931 (33)	G (paternal)	22	15
rs4750491 (35)	T (maternal)	9	21
rs1090705 (36)	G (paternal)	25	13
rs9663645 (36)	T (maternal)	11	23
rs200488869 (36)	T (maternal)	11	23

### Interaction between environmental factors and POE on T2D

**Figure 2:**
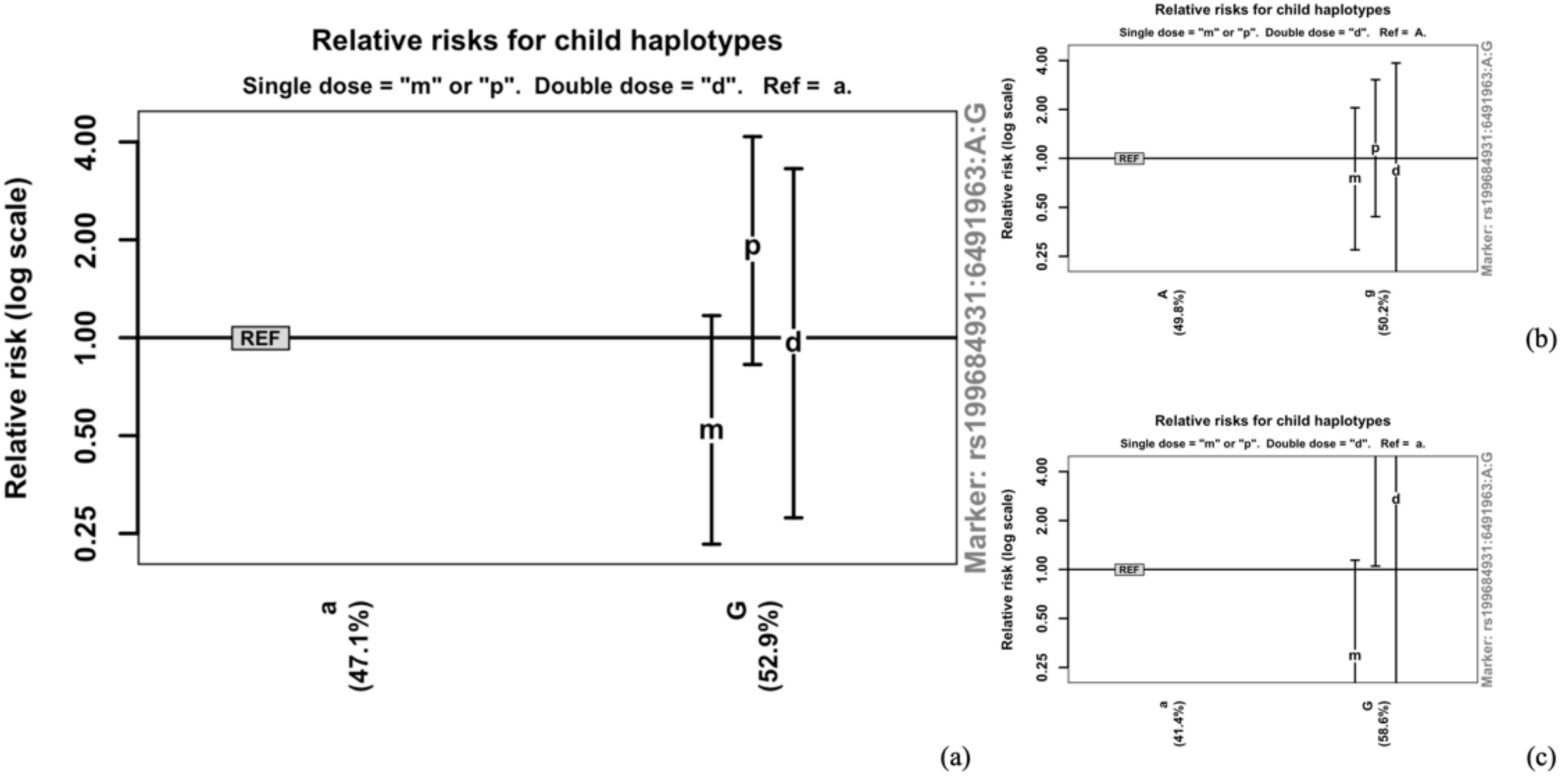
Parent-of-origin effects (POEs) stratified by BMI. **(a)** Total effects of POE of rs199684931 on T2D. This plot shows the total effects of POEs. The relative risk (RR) is displayed with 95% confidence intervals. **(b)** POE of rs199684931 at BMI <28 kg/m^2^. The effects of POE for rs199684931 are stratified based on BMI levels less than 28 kg/m^2^, showing the RR with 95% CIs in this category. **(c)** POE of rs199684931 at BMI ≥28 kg/m^2^. The effects of POE for rs199684931 are stratified based on BMI levels greater than or equal to 28 kg/m^2^, showing the RR with 95% CIs in this category.

The potential POEs of individual SNPs within the PIP2 pathway genes on T2D were analyzed under stratification by smoking status, drinking status, and BMI, respectively. All 214 SNPs identified as nominally significant for POE on T2D were further examined for interaction effects. Among these, five SNPs from *PRKCQ* exhibited significant POE–BMI interaction (**Table 3**; **Figure 2** for rs199684931 and **Supplementary Figure S2** for the other four SNPs), whereas none achieved significant interaction levels when stratified by smoking or drinking status. Notably, none of the five SNPs that exhibited nominally significant POE–BMI interaction remained statistically significant after FDR correction. Maternal effects of POE tended to be lower in rs199684931 and rs1090705 in higher BMI groups compared with lower BMI groups, while maternal effects of POE were higher in higher BMI groups for rs4750491, rs9663645, and rs200488869. Certainly, it is crucial to note that some of the CIs for these interaction effects were exceedingly wide (e.g., 3.31–393 for rs4750491 at high BMI population), indicating substantial statistical uncertainty. Therefore, these specific estimates of effect magnitude and direction should be interpreted with caution.

**Table 3:** Results of POE–BMI interaction analysis on T2D.

SNP	BMI strata (kg/m^2^)	POE		*P*_interaction_
		RR_m_/RR_p_	*P*	
rs199684931	>=28	0.03 (0.003–0.35)	**<0.01**	**0.03**
	<28	0.66 (0.18–2.31)	0.51	
	Overall	0.28 (0.10–0.78)	**0.01**	
rs4750491	>=28	36.40 (3.31–393)	**<0.01**	**0.04**
	<28	2.23 (0.68–7.10)	0.18	
	Overall	4.67 (1.71–12.30)	**<0.01**	
rs1090705	>=28	0.03 (0.003–0.31)	**<0.01**	**0.04**
	<28	0.44 (0.13–1.56)	0.20	
	Overall	0.21 (0.08–0.58)	**<0.01**	
rs9663645	>=28	36.40 (3.31–392)	**<0.01**	**0.04**
	<28	2.25 (0.64–7.58)	0.20	
	Overall	4.75 (1.72–12.80)	**<0.01**	
rs200488869	>=28	36.30 (3.34–396)	**<0.01**	**0.04**
	<28	2.25 (0.64–7.58)	0.20	
	Overall	4.75 (1.72–12.80)	**<0.01**	

Bold font symbolized results with statistical significance.

### Intersection between significant POE SNPs in the PIP2 pathway and DNA methylation

Among the evidence published earlier based on Han Chinese, 284,128 were identified as mQTL loci (34). Intersected with the 214 significant SNPs in the POE above, a total of 72 SNPs remained, suggesting that 1/3 of the POEs caused by them may be concluded via DNA methylation. These 72 SNPs were from *PRKCQ*, *PRKCE*, *MGLL*, *DGKB*, *DGKLB*, *ITPR1*, *DGKQ*, and *ITPR2* (**Supplementary Table S3**).

In the subset of 99 participants, to discover the association between the CpG sites near the top 10 significant SNPs with T2D, 11 CpG sites were found nominally significant (**Supplementary Table S4**). For example, the methylation level of CpG site cg01133103 near rs6798160 on the *ITPR1* gene exhibited a negative association with T2D.

### Enrichment analysis by Gene Ontology

Gene Ontology (GO) pathway enrichment analysis was conducted for all genes corresponding to SNPs with *P* < 0.05 in the POE analysis above, including *PRKCQ*, *DGKB*, *MGLL*, *PRKCE*, *DGKH*, *DAGLB*, *ITPR1*, *ITPR2*, *DGKQ*, *PRKCH*, *RASGRP2*, *DGKD*, and *DGKG.* The results (**Supplementary Figure S3**) show that the genes are primarily involved in lipid metabolism and signaling, as indicated by terms such as ATP-dependent DAG kinase activity, lipid binding, and acylglycerol lipase activity. Also, biological processes, including DAG metabolic processes and protein kinase C signaling pathways, are related to the genes. Furthermore, the proteins coded by these genes may localize to the cellular components, including the plasma membrane and the platelet dense tubular network membrane.

### Sensitivity analysis

A sensitivity analysis has been conducted by classifying offspring sex. Thirty-one families with male offspring and 23 families with female offspring were included in the analysis. The results of the abovementioned top 10 significant SNPs remained generally robust after the classification; however, some results were not significant due to the limited sample size. Detailed results are provided in **Supplementary Tables S5, S6**.

## Discussion

The current study examined the POE of SNPs from the PIP2 pathway on T2D and explored the potential environmental modifiers in a Chinese family-based study. The study highlighted two main findings: First, there are POEs of SNPs in the PIP2 pathway on T2D. Second, among the specific five SNPs, BMI and POE have an interaction effect on T2D.

In this study, 214 SNPs in the PIP2 pathway genes showed *P* < 0.05 of POE on T2D, covering *PRKCQ*, *DGKB*, *MGLL*, *PRKCE*, *DGKH*, *DAGLB*, *ITPR1*, *ITPR2*, *DGKQ*, *PRKCH*, *RASGRP2*, *DGKD*, and *DGKG. PRKCQ*, encoding a member of the protein kinase C (PKC) family, is associated with type 1 diabetes by a large GWAS evidence (36), which could control biological processes involving T-cell integration and CD28 signaling (37). A genetic study (38) showed that *DGKB* involved loci associated with insulin secretion and processing, potentially leading to insulin resistance, and further connected to T2D. Additionally, *MGLL* and *PRKCE* encode an enzyme hydrolyzing monoglycerides into free fatty acids and the epsilon isoform of the PKC family, both playing a significant role in lipid metabolism, indirectly associated with T2D. The genes containing the nominally significant SNPs of POE on T2D play critical roles in pathways involving DAG and protein kinase C, according to the enrichment analysis, supporting the crucial roles of these genes in lipid metabolism. Among them, the observed POE for SNPs in the *PRKCQ* gene is intriguing and may be rooted in its established biological function and potential for epigenetic regulation. Commonly known as a key regulator of T-cell activation and NF-κB signaling (39), protein kinase C (PKC)-theta sits at the nexus of immune and metabolic pathways relevant to T2D. PKC-theta is a kinase encoded by the *PRKCQ* gene, with the protein knockout decreasing the secretion of IL-10 and reducing β-cell mass and insulin secretion in pancreatic islets (40, 41). Aligning with the former report, restrictions of the maternal PKC protein might lead to insulin resistance (42). However, the observed different direction of paternal and maternal effects needs to be validated and elucidated in the future.

Although the imprinting of *PRKCQ* has not been definitively confirmed, the phenomenon is known in other PKC family members, and epigenetic mechanisms are fundamentally involved in establishing and maintaining such parent-of-origin-specific expression patterns. We postulate that the *PRKCQ* locus may harbor a complex regulatory landscape where specific SNPs interact with parental origin to fine-tune gene expression in a cell-type-specific manner, potentially modulated by environmental factors like BMI. This speculative model provides a testable hypothesis for future functional studies aimed at directly measuring allele-specific expression and methylation in relevant human tissues.

Providing indirect evidence of the overlap with mQTLs, the underlying mechanism behind the POE of these SNPs on T2D may be related to DNA methylation and imprinted genes, as in the molecular aspect (7, 32, 33). Prior studies have provided evidence on DNA methylation with lipid metabolism: in *KCNQ1* (10), DNA methylation is inversely associated with insulin sensitivity (10), and in *IGF2*/*H19* (43), DNA methylation is positively correlated with subcutaneous adiposity. Also, SNPs in *KCNQ1 (*9) at the imprinted 11p15.5 region only confer the risk of T2D when maternally inherited (9), highlighting a POE on T2D. Furthermore, non-imprinting areas, which are associated with endocrine metabolism, also have potential relations with T2D through POE, like *PDX1 (*44) and *INS* (45), whose expressions are influenced by the difference of DNA methylation in the pancreatic B-cell genome, affecting the blood sugar homeostasis. In the PIP2 pathway, the imprinted gene, *RASGRP1*, is reported to be associated with T2D and glycemic control in Asian populations (46, 47).

Obesity is recognized as a significant risk factor for T2D, with studies proving that stratifying T2D cases by BMI may help identify more genetic risk variants (48). Previous research had discovered the relationship between parental BMI and gene imprinting (49, 50), which might indirectly modify the POE. In this study, rs199684931 and the other four SNPs from *PRKCQ* were nominally significant when testing the POE stratified by BMI levels, displaying potentially different POEs on T2D across BMI stratifications. However, the wide range of the point estimates underscores the urgent need to explore this modifier of POE in larger genetic studies. A population-based study by Southeast Asians (51) also observed a maternal history significantly associated with greater BMI levels. As shown by the results, the maternal effects of POE stratified at higher BMI levels tended to be at lower risk levels in rs199684931 and rs1090705. In contrast, there were higher risks in rs4750491, rs9663645, and rs200488869. All these SNPs are novel and are reported to be associated with T2D. A large meta-analysis (52) covering over 97,000 participants illustrated that BMI could be an effective stratified factor for polygenic risk prediction of T2D, suggesting the potential modifying role of BMI on T2D heritability.

Consistent with former studies, environmental factors such as exposure to smoking and BMI could alter the epigenetic landscape by acting on the regulation of DNA methylation, potentially further amplifying the effect of specific genes on T2D (52–54). Smoking (55) and drinking (56) status of parents are also associated with offspring epigenetic modifications at early life stages. However, whether these associations remain or whether the offspring’s behaviors have the same association at later life stages with chronic diseases needs to be explored.

Several strengths support this study: First, the family-based triad design is unique for POE analysis, as it effectively controls the genetic background of participants and other possible confounding factors. Second, the POE analysis includes stratification by environmental factors, which provides a novel phenotypical aspect to explore the POE mechanism. Limitations exist, including the race of the participants being limited to Chinese. The primary limitation of this study is its modest sample size. Although the family-based design rigorously controls for population stratification, the number of case-parent triads (*n* = 54) limits the statistical power to detect genetic effects, particularly those with small effect sizes or complex interactions. Consequently, this work should be considered exploratory in nature, aiming to generate hypotheses for future investigation rather than to provide definitive conclusions. Overly strict definitions of significance could increase the likelihood of missing potential signals since T2D is an etiologically heterogeneous phenotype. Thus, we applied *P* < 0.05 as the threshold rather than multiple comparisons, considering the number of tests performed. Further studies with a larger sample are warranted to replicate our findings.

## Conclusion

This study suggested that SNPs of the PIP2 pathway genes may have POEs on T2D, and the POEs of five SNPs in the *PRKCQ* gene have potential interaction with BMI on T2D. Considering the limited sample size, future studies are needed to explore the detailed mechanisms from the molecular aspects and replicate our findings.

## Data Availability

The data analyzed in this study is subject to the following licenses/restrictions: The data presented in this study are available on request from the corresponding author. The data are not publicly available due to the policy of the Ethics Committee of the Peking University Health Science Center. Requests to access these datasets should be directed to Mengying Wang, mywang@bjmu.edu.cn.
