# Supplementary material for: Parent-of-origin effects of phosphatidyl inositol-bisphosphate hydrolysis pathway genes on type 2 diabetes and the modification effect by obesity
Source: Front Endocrinol (Lausanne). 2025 Nov 27;16:1705584. doi: 10.3389/fendo.2025.1705584 (PMC12695578; doi:10.3389/fendo.2025.1705584)
Supplement: Supplementary file 1 [file DataSheet1.docx]

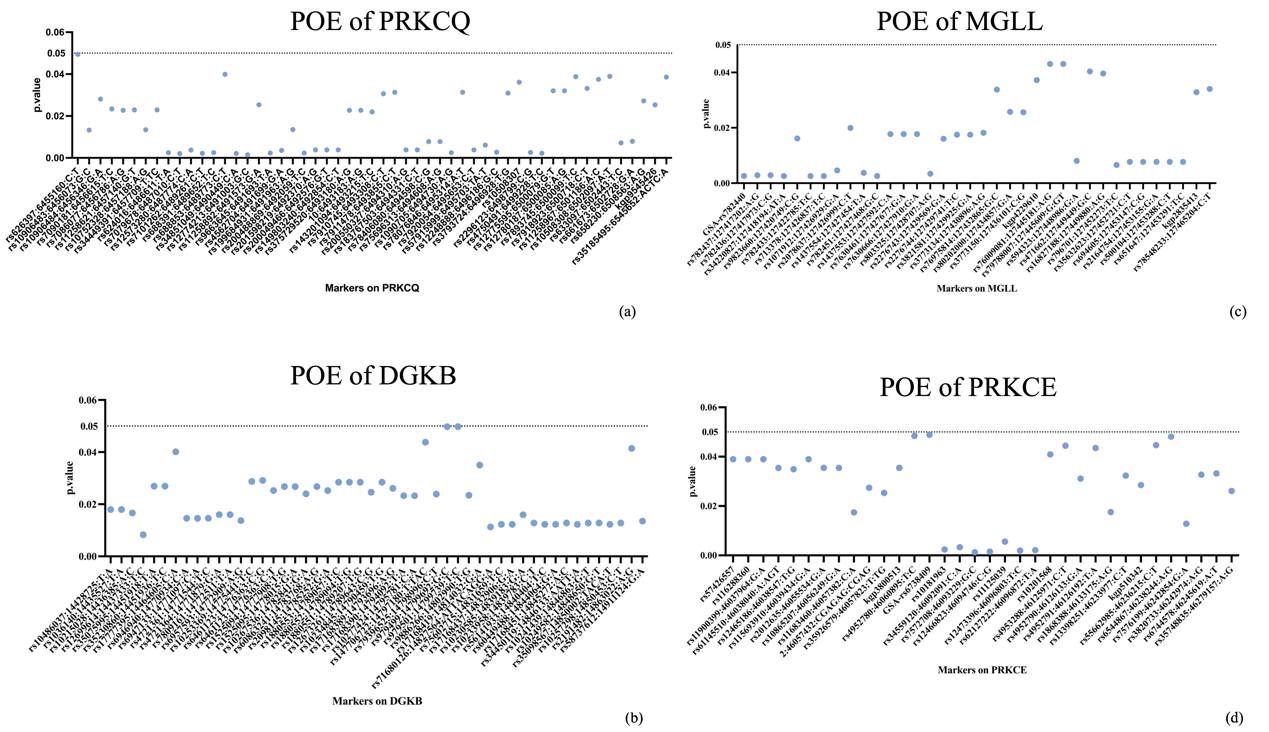


**Supplementary Figure 1 SNPs in the PIP2 pathway genes achieved a significant level (*P*<0.05) in POE analysis**

The SNPs in each gene went through HaplinSlide code, *P*<0.05 was recognized as significant. Figure 2a: *PRKCQ*; 2b: *DGKB*; 2c: *MGLL*; 2d: *PRKCE*.


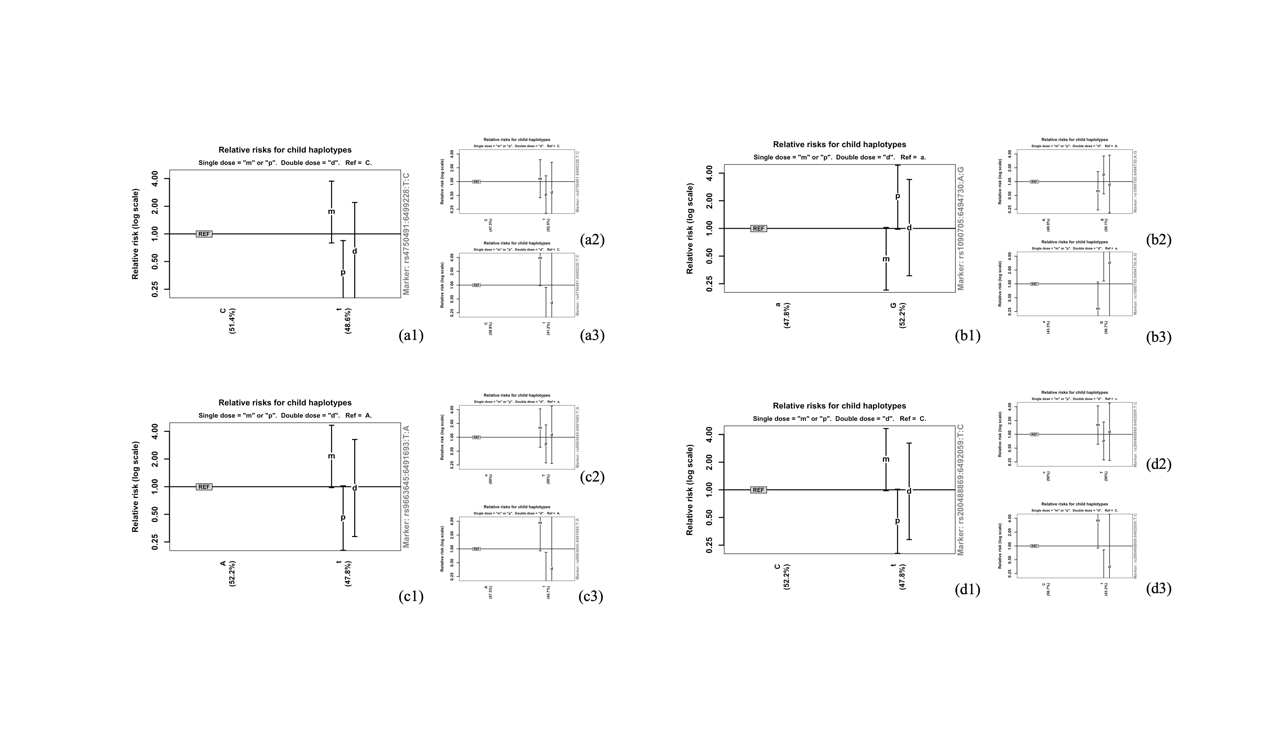


**Supplementary Figure 2 POE stratified by BMI of 4 SNPs**

**Figure a1:** Total effects of POE of rs4750491. This plot shows the total effects of parent-of-origin effects (POE) on rs4750491in the child phenotype. The relative risk (RR) is displayed with 95% confidence intervals.

**Figure a2:** POE of rs4750491at BMI < 28. The effects of POE for rs4750491are stratified based on BMI levels less than 28, showing the RR with 95% CIs in this category.

**Figure a3:** POE of rs4750491at BMI ≥ 28. The effects of POE for rs4750491are stratified based on BMI levels greater than or equal to 28, showing the RR with 95%

**Figures b, c, and d** likewise represent the results for rs1090705, rs9663645, and rs200488869, respectively.


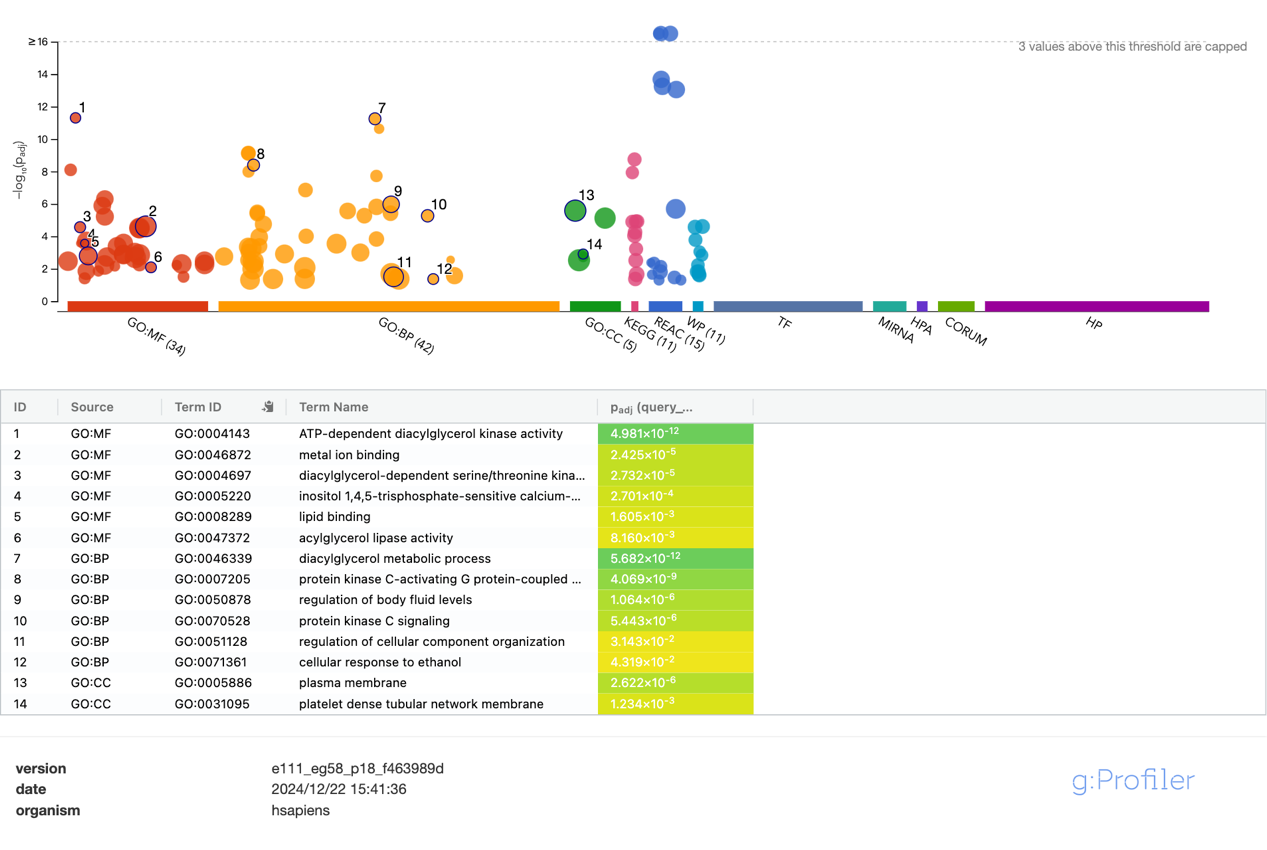


**Supplementary Figure 3 Enrichment analysis results by Gene Ontology**

**Supplementary Table 1 Characteristics of SNPs analyzed in the PIP2 pathway**

| Chrom  some | Gene | Covering area  (GRCh37.p13) | SNPs | | |
| --- | --- | --- | --- | --- | --- |
|  |  |  | Included in | Bonferroni criteria |  |
| 20 | *ABHD12* | 25275379 ~25371471 | 210 | 2.38*10^-4^ |  |
| 3 | *ABHD6* | 58223519 ~58280461 | 91 | 5.49*10^-4^ |  |
| 11 | *DAGLA* | 61447863 ~61514473 | 112 | 4.46*10^-4^ |  |
| 7 | *DAGLB* | 6448760 ~6487585 | 144 | 3.47*10^-4^ |  |
| 12 | *DGKA* | 56321100 ~56347807 | 13 | 3.85*10^-3^ |  |
| 7 | *DGKB* | 14184674 ~15014483 | 1527 | 3.27*10^-5^ |  |
| 2 | *DGKD* | 234263140 ~234380744 | 162 | 3.09*10^-4^ |  |
| 17 | *DGKE* | 54911512 ~54946928 | 70 | 7.14*10^-4^ |  |
| 3 | *DGKG* | 185864990 ~186080023 | 216 | 2.31*10^-4^ |  |
| 13 | *DGKH* | 42614206 ~42830720 | 729 | 6.86*10^-5^ |  |
| 7 | *DGKI* | 137065783 ~137531720 | 875 | 5.71*10^-5^ |  |
| X | *DGKK* | 50108408 ~50213824 | 15 | 3.33*10^-3^ |  |
| 4 | *DGKQ* | 952675 ~967357 | 32 | 1.56*10^-3^ |  |
| 11 | *DGKZ* | 46354476 ~46402104 | 43 | 1.16*10^-3^ |  |
| 3 | *ITPR1* | 4535032 ~4889190 | 540 | 9.26*10^-5^ |  |
| 12 | *ITPR2* | 26488285 ~26986127 | 1016 | 4.92*10^-5^ |  |
| 6 | *ITPR3* | 33589099 ~33664339 | 267 | 1.87*10^-4^ |  |
| 3 | *MGLL* | 127407909 ~127542028 | 338 | 1.48*10^-4^ |  |
| 3 | *PRKCD* | 53195225 ~53226733 | 58 | 8.62*10^-4^ |  |
| 2 | *PRKCE* | 45878814 ~46415129 | 962 | 5.20*10^-5^ |  |
| 14 | *PRKCH* | 61788297 ~62017694 | 532 | 9.40*10^-5^ |  |
| 10 | *PRKCQ* | 6436059 ~ 6622608 | 378 | 1.32*10^-4^ |  |
| 15 | *RASGRP1* | 38780304 ~38857015 | 44 | 1.13*10^-3^ |  |
| 11 | *RASGRP2* | 64494383 ~64512928 | 19 | 2.63*10^-3^ |  |
| 4 | *TRPC3* | 122795636 ~122873215 | 52 | 9.62*10^-4^ |  |
| 11 | *TRPC6* | 101322295 ~101454738 | 469 | 1.07*10^-4^ |  |
| 5 | *TRPC7* | 135548433 ~135701233 | 277 | 1.81*10^-4^ |  |

**Supplementary Table 2 Characteristics of genes and SNPs in the PIP2 pathway genes for POE analysis**

| Gene | Number of SNPs | |  |
| --- | --- | --- | --- |
|  | Significant after Bonferroni correction | Significant by 0.05 | |
| *ABHD12* | 0 | 0 | |
| *ABHD6* | 0 | 0 | |
| *DAGLA* | 0 | 0 | |
| *DAGLB* | 0 | 7 | |
| *DGKA* | 0 | 0 | |
| *DGKB* | 0 | 50 | |
| *DGKD* | 0 | 1 | |
| *DGKE* | 0 | 0 | |
| *DGKG* | 0 | 1 | |
| *DGKH* | 0 | 8 | |
| *DGKI* | 0 | 0 | |
| *DGKK* | 0 | 0 | |
| *DGKQ* | 0 | 4 | |
| *DGKZ* | 0 | 0 | |
| *ITPR1* | 0 | 7 | |
| *ITPR2* | 0 | 5 | |
| *ITPR3* | 0 | 0 | |
| *MGLL* | 0 | 36 | |
| *PRKCD* | 0 | 0 | |
| *PRKCE* | 0 | 34 | |
| *PRKCH* | 0 | 4 | |
| *PRKCQ* | 0 | 53 | |
| *RASGRP1* | 0 | 0 | |
| *RASGRP2* | 0 | 4 | |
| *TRPC3* | 0 | 0 | |
| *TRPC6* | 0 | 0 | |
| *TRPC7* | 0 | 0 | |

**Supplementary Table 3 SNP list of intersection between significant POE and DNA methylation**

| SNP | Gene |
| --- | --- |
| rs57426557 | *PRKCE* |
| rs4953288 | *PRKCE* |
| rs782433 | *MGLL* |
| rs2276744 | *MGLL* |
| rs702485 | *DAGLB* |
| rs1880553 | *DGKB* |
| rs979501 | *DGKB* |
| rs4562704 | *PRKCQ* |
| rs7911478 | *PRKCQ* |
| rs4750491 | *PRKCQ* |
| rs116288360 | *PRKCE* |
| rs4952780 | *PRKCE* |
| rs4952790 | *PRKCE* |
| rs713378 | *MGLL* |
| rs3821581 | *MGLL* |
| rs836562 | *DAGLB* |
| rs1880552 | *DGKB* |
| rs9886266 | *DGKB* |
| rs200488869 | *PRKCQ* |
| rs200550327 | *PRKCQ* |
| rs7918923 | *PRKCQ* |
| rs11900399 | *PRKCE* |
| rs6738409 | *PRKCE* |
| rs4074087 | *ITPR1* |
| rs1437553 | *MGLL* |
| rs80202000 | *MGLL* |
| rs836500 | *DAGLB* |
| rs1880551 | *DGKB* |
| rs17168419 | *DGKB* |
| rs201598219 | *PRKCQ* |
| rs183767503 | *PRKCQ* |
| rs11258967 | *PRKCQ* |
| rs12465186 | *PRKCE* |
| rs10181963 | *PRKCE* |
| rs3805012 | *ITPR1* |
| rs7630461 | *MGLL* |
| rs76009081 | *MGLL* |
| rs836510 | *DAGLB* |
| rs10234845 | *DGKB* |
| rs12573280 | *PRKCQ* |
| rs200049466 | *PRKCQ* |
| rs1090705 | *PRKCQ* |
| rs10842798 | *ITPR2* |
| rs2012635 | *PRKCE* |
| rs11125039 | *PRKCE* |
| rs6798160 | *ITPR1* |
| rs7630666 | *MGLL* |
| rs78548233 | *MGLL* |
| rs836540 | *DAGLB* |
| rs17168389 | *DGKB* |
| rs7101262 | *PRKCQ* |
| rs149803240 | *PRKCQ* |
| rs7920054 | *PRKCQ* |
| rs10865207 | *PRKCE* |
| rs12473396 | *PRKCE* |
| rs782440 | *MGLL* |
| rs803325 | *MGLL* |
| rs28671147 | *DGKQ* |
| rs35230860 | *DGKB* |
| rs10277424 | *DGKB* |
| rs688391 | *PRKCQ* |
| rs3793724 | *PRKCQ* |
| rs11683460 | *PRKCE* |
| rs10201568 | *PRKCE* |
| rs782437 | *MGLL* |
| rs2276743 | *MGLL* |
| rs6599390 | *DGKQ* |
| rs10267536 | *DGKB* |
| rs79396525 | *DGKB* |
| rs34851049 | *PRKCQ* |
| rs7910745 | *PRKCQ* |
| rs10508307 | *PRKCQ* |

**Supplementary Table 4 Association between the CpG sites near the top 10 significant SNPs with T2D**

| CpG site | Position | Gene | Beta | SD | P value |
| --- | --- | --- | --- | --- | --- |
| cg01133103 | 3: 4765015 | *ITPR1* | -3.86 | 1.33 | 0.005 |
| cg14651327 | 2: 45998165 | *PRKCE* | 3.46 | 1.31 | 0.010 |
| cg03630683 | 3: 4534997 | *ITPR1* | 18.70 | 7.51 | 0.014 |
| cg01538573 | 3: 4744662 | *ITPR1* | -3.19 | 1.35 | 0.020 |
| cg12187810 | 2: 45917640 | *PRKCE* | -2.02 | 0.86 | 0.021 |
| cg23251299 | 3: 4545007 | *ITPR1* | -1.83 | 0.81 | 0.027 |
| cg24820593 | 2: 46290700 | *PRKCE* | 6.08 | 2.84 | 0.035 |
| cg17278444 | 3: 4572945 | *ITPR1* | -3.38 | 1.66 | 0.044 |
| cg24319825 | 3: 4534939 | *ITPR1* | 25.03 | 12.50 | 0.048 |
| cg21046616 | 10: 6535421 | *PRKCQ* | 3.89 | 1.95 | 0.049 |
| cg03239523 | 3: 4793370 | *ITPR1* | -1.08 | 0.54 | 0.049 |

**Supplementary Table 5 The results of the most significant loci of POE on T2D, in male offspring**

| Gene | SNP | Estimate of POE, the ratio RR_m_/RR_f_ | lower | upper | *P* value of POE |
| --- | --- | --- | --- | --- | --- |
| *ITPR1* | rs6798160 | 0.07 | 0.01 | 0.44 | **0.004** |
| *PRKCE* | rs7572708 | 18.89 | 3.41 | 108.32 | **8.09**$\boldsymbol{\times}$**10^-4^** |
| *PRKCQ* | rs12774213 | 0.08 | 0.02 | 0.37 | **0.001** |
| *PRKCE* | rs12466823 | 18.78 | 3.20 | 104.59 | **0.001** |
| *PRKCE* | rs12473396 | 0.25 | 0.07 | 0.89 | **0.034** |
| *PRKCQ* | rs7079678 | 0.09 | 0.02 | 0.43 | **0.002** |
| *PRKCQ* | rs34851049 | 0.09 | 0.02 | 0.42 | **0.002** |
| *PRKCE* | rs62127222 | 0.25 | 0.07 | 0.90 | **0.036** |
| *PRKCQ* | rs7101262 | 0.09 | 0.02 | 0.43 | **0.002** |
| *PRKCQ* | rs4750491 | 7.21 | 1.89 | 26.50 | **0.003** |

**Supplementary Table 6 The results of the most significant loci of POE on T2D, in female offspring**

| Gene | SNP | Estimate of POE, the ratio RR_m_/RR_f_ | lower | upper | *P* value of POE |
| --- | --- | --- | --- | --- | --- |
| *ITPR1* | rs6798160 | 0.17 | 0.03 | 1.00 | 0.053 |
| *PRKCE* | rs7572708 | 2.02 | 0.21 | 20.17 | 0.531 |
| *PRKCQ* | rs12774213 | 0.39 | 0.09 | 1.86 | 0.232 |
| *PRKCE* | rs12466823 | 2.15 | 0.21 | 23.15 | 0.511 |
| *PRKCE* | rs12473396 | 0.11 | 0.02 | 0.69 | **0.018** |
| *PRKCQ* | rs7079678 | 0.41 | 0.08 | 1.84 | 0.241 |
| *PRKCQ* | rs34851049 | 0.39 | 0.09 | 1.86 | 0.232 |
| *PRKCE* | rs62127222 | 0.11 | 0.02 | 0.69 | **0.018** |
| *PRKCQ* | rs7101262 | 0.41 | 0.08 | 1.84 | 0.241 |
| *PRKCQ* | rs4750491 | 2.69 | 0.56 | 12.61 | 0.218 |
